# Percutaneous bone-anchored hearing implant surgery: dermatome versus linear incision technique

**DOI:** 10.1007/s00405-016-4210-3

**Published:** 2016-07-20

**Authors:** Ruben M. Strijbos, Steven J. H. Bom, Stefan Zwerver, Myrthe K. S. Hol

**Affiliations:** 1 Department of Otorhinolaryngology, Radboud University Medical Centre, Post 377, PO box 9101, 6500 HB Nijmegen, The Netherlands; 2 Department of Otorhinolaryngology, Deventer Hospital, Deventer, The Netherlands

**Keywords:** Baha, Bone-anchored hearing implant, Surgical technique, Linear incision, Dermatome technique, Soft tissue reactions

## Abstract

The objective of this historical cohort study is to identify if there are differences in soft tissue reactions and skin thickening between implantation of the percutaneous bone-anchored hearing implant (BAHI) using the dermatome or linear incision technique. All adult patients who received a BAHI between August 2005 and January 2013 were selected. One surgeon performed all procedures and only the dermatome and linear incision technique were used. A total of 132 patients/implants were included and significantly more patients with risk factors were seen in the linear incision cohort. A soft tissue reaction Holgers ≥1 was present in 18 patients (40.9 %) in the dermatome compared to 36 patients (40.9 %) in the linear incision group. A Holgers ≥2 was noticed in 9 (20.5 %) and 19 (21.6 %) patients, respectively. Skin thickening was described in 14 (31.8 %) and 11 patients (12.5 %) in, respectively, the dermatome and linear incision cohort, which was a significant difference (*p* = 0.001). Nevertheless, therapeutic interventions were effective. In conclusion, there was no significant difference in (adverse) soft tissue reactions; however, skin thickening was more present in the dermatome technique. In addition, significantly more patients with risk factors were allocated to the linear incision technique. Based on these results, the linear incision is advocated as preferred technique.

## Introduction

Since the first implantation in 1977 by Tjellström, percutaneous bone-anchored hearing implants (BAHIs) offer an appealing solution in hearing rehabilitation for patients with a conductive or mixed hearing loss [1, 2] and single-sided deafness [3–6]. These devices stimulate the cochlea directly through the principle of bone conduction [1]. The ongoing developments in the field of bone conduction devices have led to a safe procedure of implantation with a lack of major complications [7]. However, depending on type of implant and abutment, surgical technique and postoperative care, soft tissue reactions are still occasionally a problem [7–12]. The Holgers’ classification is most commonly used to grade these soft tissue reactions [12].

The surgical procedure for implantation in adults is nowadays performed as a one-staged procedure [2]. Various surgical techniques have been developed, which started with the free retroauricular full-thickness skin graft [13] and later the pedicled grafts [14]. Over the years, the dermatome and linear incision technique have been introduced with the goal to further minimize skin problems postoperatively [15, 16]. The dermatome technique was developed to standardize the pedicled flap technique and create a thinner skin graft. A Baha dermatome is used to create a skin graft without hair follicles, which stays attached to the skin on one side. The soft tissue beneath will be removed, with the creation of a gradual slope down to the implant site. The periosteum remains intact with exception of the place of insertion of the implant [17, 18]. In the linear incision technique, a longitudinal incision of about 30 mm posterosuperiorly to the ear canal is made. The periosteum is exposed and mobilized after sharp dissection of the subcutaneous tissue. Subsequently, the implant is placed and subcutaneous tissue will be resected over an area of approximately 2 cm around the incision. In addition, the remaining periosteum is removed [19]. Recent studies show promising results in the context of surgical techniques with tissue preservation [20–23].

Based on the available literature, studies reporting about the dermatome technique show an overall higher rate of skin problems compared to studies regarding the linear incision technique and nowadays this latter technique is gaining more interest as standard of care. Nevertheless, variability in setup, follow-up and surgical techniques among these studies may influence the rate of skin complications [16]. To our knowledge, there are only two comparative studies that evaluate major postoperative complications between these two techniques: one as part of a comparison of several techniques [24] and another with a limited follow-up without using the Holgers classification [25]. The aim of the current historical cohort study is to provide more rigorous support of the superiority of the linear incision technique by directly comparing both the dermatome and linear incision technique with subcutaneous soft tissue reduction in adults. There will be an evaluation if there are differences in the presence of soft tissue reactions, as classified by the Holgers grading system, and skin thickening between these two techniques, alternatively performed by a single surgeon in a general, teaching hospital.

## Materials and methods

### Patients

All adult patients (aged 18 years or older) who received any type of percutaneous bone-anchored hearing implant (BAHI) at one large Dutch general, teaching hospital between August 2005 and January 2013 were consecutively selected from our local Bone Implant database. Indications for a percutaneous BAHI were conductive or mixed hearing loss and single-sided deafness. Eligibility criteria were: one-staged procedure, primary placement of the implant (no previous implant removal or loss) and availability of the patient’s medical chart including at least one postoperative visit at the outpatient clinic.

### Surgical techniques and post-surgery protocol

Only the dermatome technique [17] and simplified linear incision technique with subcutaneous soft tissue reduction [19] were used in the selected study period. In addition, all patients were operated on by the same surgeon (S.B.). There was preoperatively screening for an increased risk of skin flap necrosis [17]. If one or more possible risk factors were present or suspected, patients were operated with the linear incision technique. Otherwise a patient underwent generally the procedure using the dermatome technique. Risk factors were high age (75 years or older), smoking, diabetes mellitus, mental retardation or cardiovascular comorbidity [26–29].

The first postoperative visit was 1 week after surgery, when the healing cap and gauze with antibiotic ointment (only in the 41 first patients) or Mepilex foam (Mölynlycke Health Care, Gothenburg, Sweden; in the majority of patients) were removed. The wound was inspected and all patients received, conform protocol in the hospital, topical therapy with fusidic acid for 2–4 weeks. Further follow-up was after 3 weeks, 6 months and 12 months and then in principle every year. Extra appointments were arranged by patients or physicians if problems arose or depending on individual needs. During each visit, there was registration of the degree of soft tissue reaction and skin thickening. If any postoperative problems occurred, i.e. skin flap necrosis, wound dehiscence or implant loss, this was also recorded. Besides, there was registration of therapeutic interventions, if applicable. End of the follow-up was defined as the last follow-up before November 2015.

### Case analysis

All data were obtained from the local database and patient’s medical records of the aforementioned teaching hospital. The operative report was used to collect information about the surgical technique and implant type. Furthermore, the notes from the physical examination in all follow-up contacts by one of the physicians or residents were used to determine the presence of postoperative complications, skin thickening and soft tissue reactions.

The postoperative complications were divided into skin flap necrosis, wound dehiscence or implant loss. Skin flap necrosis was further split in minor, medium or major, which indicated, respectively, a non-vital skin flap of less than 25, 25–50 % or more than 50 % of the total flap [17]. Wound dehiscence was subdivided into dehiscence without need for surgical intervention versus dehiscence which required a free skin graft. Finally, in case of implant loss there was registration of the cause.

The skin was described as low or thickened. The term skin thickening was defined as (partially) high skin around the abutment or soft tissue overgrowth. The possible therapeutic intervention was corticosteroid injection with triamcinolone acetonide, otherwise an extended abutment could be placed or eventually surgical soft tissue revision might be considered.

The soft tissue reactions were graded according to the Holgers classification [12]. A distinction was made between soft tissue reactions in general and adverse soft tissue reactions, because of the clinical implications of the latter (i.e. indication for (topical) treatment). An adverse soft tissue reaction was defined as a Holgers 2 or higher and a soft tissue reaction as a Holgers 1 or higher. Besides, if the Holgers notation was missing but there was notation of redness, swelling, moistness and/or granulation, this was interpreted as the presence of a soft tissue reaction. No notation of signs of inflammation in the physical examination was considered as a Holgers grade 0, i.e. the absence of soft tissue reaction.

Finally, the background characteristics gender, body mass index, diabetes mellitus, mental retardation, smoking and cardiovascular comorbidity were registered, following recent studies focusing on identification of these comorbidities as (potential) risk factor for soft tissue reactions or implant loss [8, 30–34]. In addition, some characteristics may be associated with skin flap necrosis or impaired wound healing [26–28].

### Statistical analysis

A comparison of background characteristics was performed using a Student’s *t* test if there was a normal distribution; otherwise, a Mann–Whitney *U* test was performed. The Kolmogorov–Smirnov test was used to determine whether the criteria for normal distribution were met. Chi-square test was performed if the outcome was a proportion.

In the context of the presence of skin thickening and (adverse) soft tissue reactions, there were survival curves calculated using the Kaplan–Meier method. The log-rank test was executed to identify differences between these curves. The level of significance applied was *p* = 0.05. All our analyses were performed using Statistical Package for Social Sciences (IBM SPSS Statistics for Windows, Armonk, NY; IBM Corp), version 22.0.

## Results

### Patients

In the period from August 2005 until January 2013, a total of 146 implants were placed. A cohort of 132 implants met the eligibility criteria, because 14 implants were excluded: 10 implants were placed in children (aged younger than 18 years) and 4 implants had no initial placement. Since none of these implants were placed bilaterally, the cohort consisted also of 132 patients. A total of 44 patients were operated using the dermatome technique with a mean age of 50.3 years (range 26–72, SD ±12.3) and median follow-up of 40.5 months (interquartile range (IQR) 22.5–72.25). The linear incision group consisted of 88 patients with a mean age of 59.3 years (range 22–89, SD ±14.3) and median follow-up of 56.5 months (IQR 29.5–89.75).

All the baseline patient characteristics are summarized in Table 1. As mentioned, patients were preoperatively screened for an increased risk of skin flap necrosis and underwent in general the linear incision technique if one or more possible risk factors were present. This explains the significant difference found in age (*p* = 0.001), diabetes mellitus (*p* = 0.039), cardiovascular comorbidity (*p* = 0.036) and smoking (*p* = 0.031) between the groups. Table 2 shows the surgical characteristics. In addition, only 5.5 and 6.0 mm (and no extended) abutments were used for previous generation Cochlear, respectively, all other implants. Moreover, significantly more previous generation implants were placed in the linear incision cohort (*p* = 0.033).

**Table 1:** Summary of the patient characteristics

	Dermatome		Linear incision		*P* values
	*n*	%	*n*	%	
Total patients/implants	44	100	88	100	
Gender					
Male	20	45.5	53	60.2	0.108
Female	24	54.5	35	39.8	
Age at surgery					
Mean (years) [±SD]	50.3 [12.3]		59.3 [14.3]		0.001*
Range (years)	26–72		22–89		
Aetiology of hearing loss					
Conductive/mixed hearing loss	34	77.3	83	94.3	
Single-sided deafness	10	22.7	5	5.7	
Comorbidity factor					
Mean body mass index (kg/m^2^) [±SD]	26.9 [4.4]		27.1 [4.4]		0.816
Diabetes mellitus	1	2.3	12	13.6	0.039*
Cardiovascular comorbidity	18	40.9	53	60.2	0.036*
Mental retardation	0	0	5	5.7	0.107
Smoking	4	9.1	21	23.9	0.031*

* Significant difference (*p* < 0.05)

**Table 2:** Summary of the surgical characteristics

Characteristics	Dermatome		Linear incision	
	*n*	%	*n*	%
Follow-up				
Median (months)	40.5		56.5	
Interquartile range (months)	22.5–72.25		29.5–89.75	
Side				
Right	23	52.3	46	52.3
Left	21	47.7	42	47.7
Implant length				
3 mm Cochlear	0	0	5	5.7
4 mm Cochlear	36	81.8	71	80.7
3 mm Oticon	0	0	1	1.1
4 mm Oticon	8	18.2	11	12.5
Implant type				
Previous generation Cochlear (“flange fixture”)	25	56.8	66	75
BIA300	11	25	10	11.4
Ponto regular	8	18.2	12	13.6
Bottom				
Bone	33	75.0	63	71.6
Dura	8	18.2	19	21.6
Bone/dura	3	6.8	6	6.8

### Postoperative complications

Skin flap necrosis was noticed only in the dermatome technique. Minor skin flap necrosis was seen in three patients (6.8 %) and medium skin flap necrosis in one patient (2.3 %). None of these cases required surgical intervention. In addition, no patient developed major skin flap necrosis. Dehiscence of the surgical wound was only seen in the linear incision technique. In 26 patients, registration of dehiscence was without need of surgical intervention (29.5 %) and in two patients the severity required a free skin graft (2.3 %). One of these patients had multiple risk factors for impaired wound healing; the other patient had postoperative persistent blood clots in the wound because of dysregulated coagulation (which impaired closure of the dehiscence).

During complete follow-up, four implants were lost which were all previous generation implants (Cochlear flange fixture, 4 mm) and placed according to the linear incision technique. All of these implants were lost after more than 6 years of follow-up (74, 78, 84 and 89 months). Two implants were lost spontaneously after a distinct period with pain, one implant was lost presumptively after a peri-implantitis and one implant was lost due to trauma. No implant was lost because of a Holgers grade 4.

### Skin thickening

**Fig. 1:**
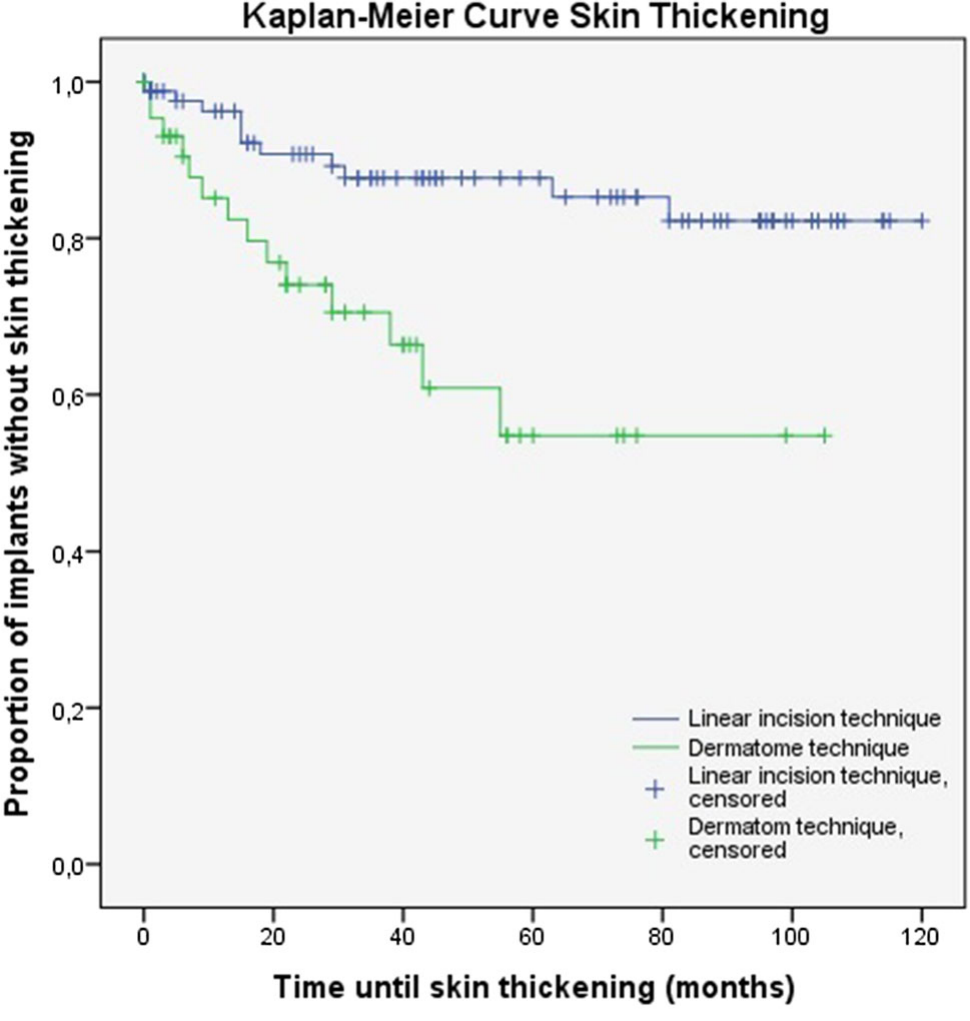
The Kaplan–Meier analysis for skin thickening (*p* = 0.001)

The presence of skin thickening was described in 14 patients (31.8 %) in the dermatome group and 11 patients (12.5 %) in the group which were operated using the linear incision technique. Nevertheless, soft tissue overgrowth was not recorded during the entire follow-up. The Kaplan–Meier curves are shown in Fig. 1. These curves are showing the probability of surviving, i.e. not encountering the condition of skin thickening, in a given length of time for patients in the different cohorts. The presence of skin thickening was significantly higher in the dermatome cohort (*p* = 0.001). In addition, Table 3 shows the therapeutic interventions in patients with skin thickening. No intervention was necessary in three patients. All other patients received triamcinolone acetonide injection and/or a higher abutment. Soft tissue reduction was performed in two patients. The therapeutic interventions were eventually effective in all cases.

**Table 3:** Overview of the different therapeutic interventions for skin thickening and how often these procedures had to be performed in every patient

	Dermatome		Linear incision	
	*n*	%	*n*	%
Number of patients with skin thickening	14	100	11	100
Number of triamcinolone acetonide injections				
0	1	7.1	3	27.3
1–2	5	35.7	5	45.5
3–5	3	21.4	2	18.2
6–10	5	35.7	1	9.1
Number of abutment changes				
0	8	57.1	8	72.7
1	6	42.9	1	9.1
2	0	0	2	18.2
Number of soft tissue reductions				
0	13	92.9	10	90.9
1	1	7.1	0	0
2	0	0	1	9.1

### Soft tissue reactions

**Fig. 2:**
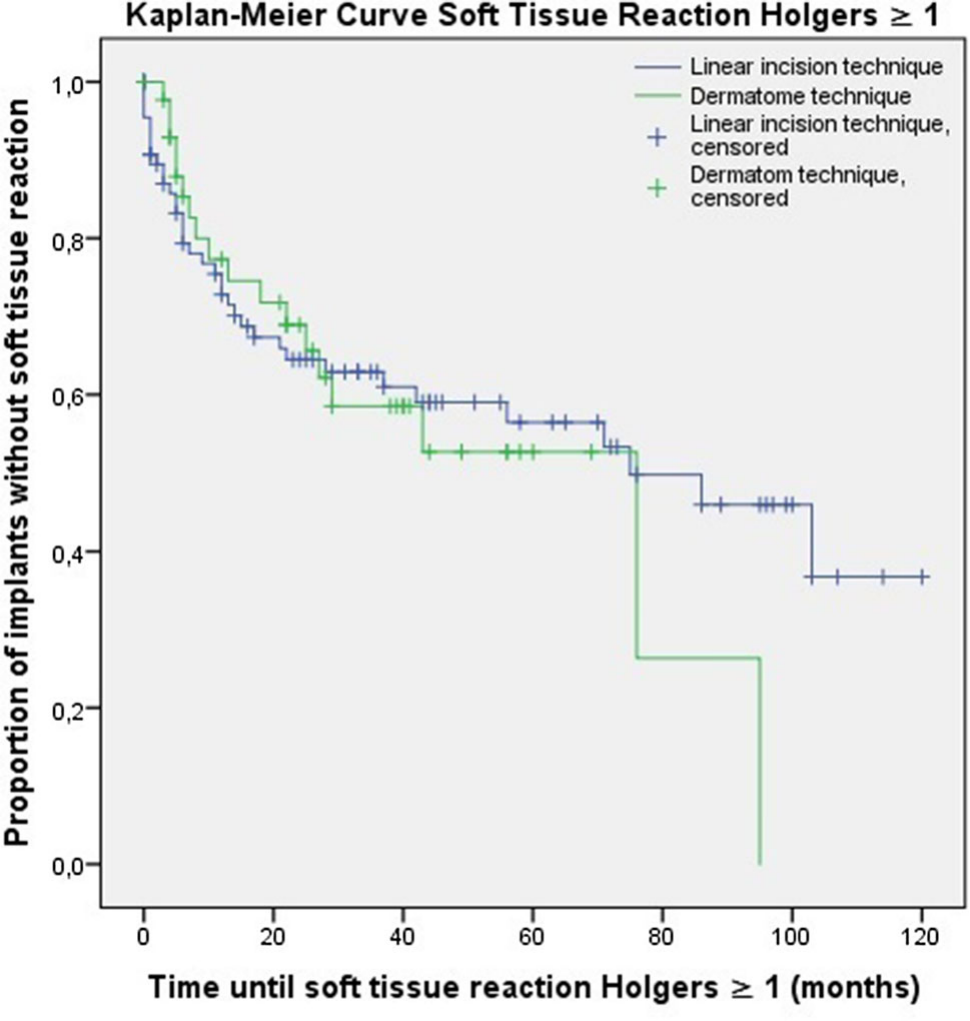
The Kaplan–Meier analysis for soft tissue reaction Holgers ≥1 (*p* = 0.710)

**Fig. 3:**
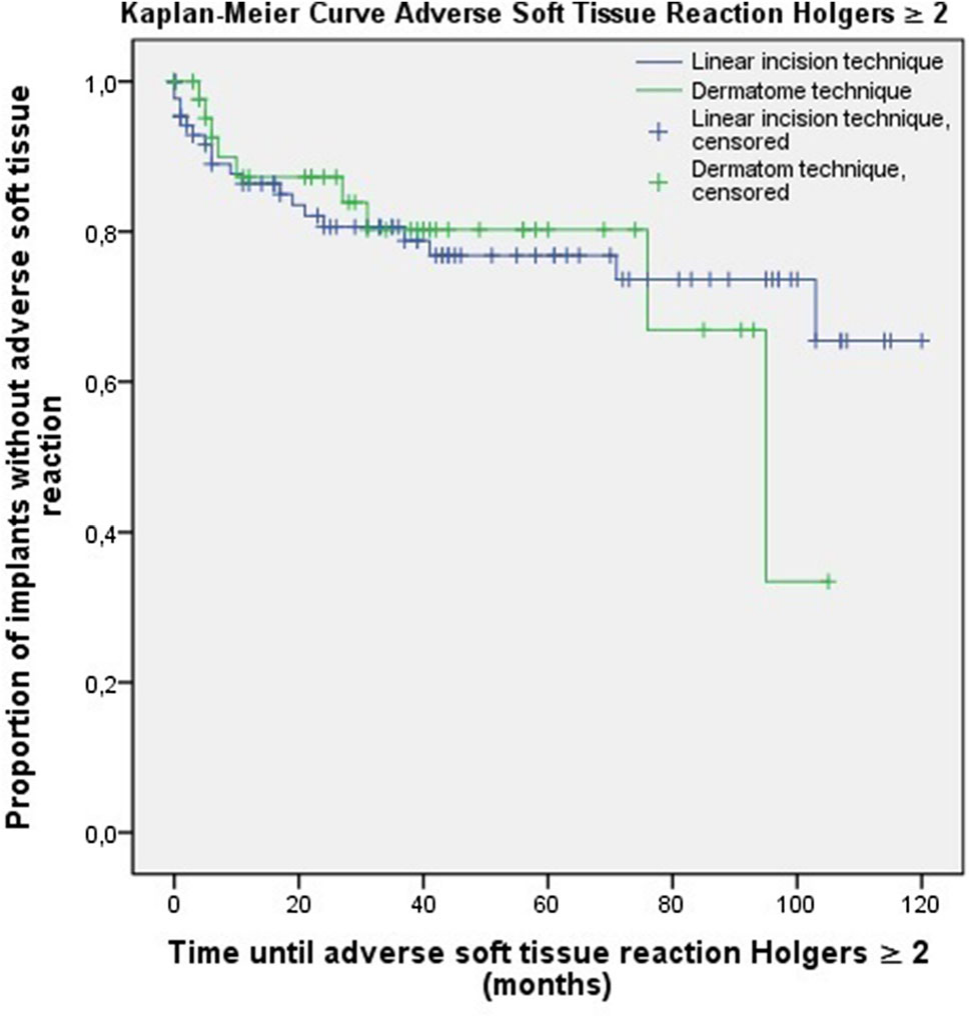
The Kaplan–Meier analysis for adverse soft tissue reaction Holgers ≥2 (*p* = 0.925)

In the group of patients operated with the dermatome technique, a soft tissue reaction (i.e. Holgers ≥1) was noticed in 18 persons (40.9 %) compared to 36 persons (40.9 %) in the group of the linear incision technique. Adverse soft tissue reactions (i.e. Holgers ≥2) were noticed in 9 patients (20.5 %) who underwent the procedure with the dermatome. In comparison, 19 patients (21.6 %) in the group of the linear incision technique encountered an adverse soft tissue reaction. For these two outcomes measures, the Kaplan–Meier method was used to calculate survival curves (Figs. 2, 3). No significant differences were found between the dermatome and linear incision technique for the presence of both soft tissue reactions (*p* = 0.710) and adverse soft tissue reactions (*p* = 0.925).

Due to the aforementioned significantly higher rate of previous generation implants in the linear incision group, a statistical subanalysis was performed for the soft tissue reactions and skin thickening according to implant type (i.e. previous generation bone implants (“flange fixture”) versus the more recent BIA300 and Ponto Regular implants). The percentage of implants encountering skin thickening, Holgers ≥1 and Holgers ≥2 was, respectively, 24.2, 48.4 and 28.6 % in the previous generation bone implant group. In the group of BIA300 and Ponto Regular implants these percentages were, respectively, 7.3, 24.4 and 4.9 %. A Kaplan–Meier analysis with log-rank test revealed that the difference in skin thickening (*p* = 0.119) and soft tissue reactions Holgers ≥1 (*p* = 0.120) was not significant. Nevertheless, significantly more adverse soft tissue reactions Holgers ≥2 were encountered in the previous generation implants (*p* = 0.020).

A subanalysis of patients without any possible risk factors for skin problems could not be performed due to a too low number of eligible patients in both cohorts for comparison.

## Discussion

In this retrospective cohort study, 132 implants were studied in 132 patients with a total median follow-up time of 47.5 months (IQR 26.0–84.75). There were no statistically significant differences found in the presence of soft tissue reactions or adverse soft tissue reactions between patients who underwent surgery with the dermatome technique and patients operated with the linear incision technique in the current set up. Skin thickening was significantly more encountered in the dermatome cohort, but could be treated successfully.

Over the last decade, several developments and improvements have been made in implant types, sound processors and surgical techniques. In the field of the latter, both the dermatome and linear incision technique became popular in many centres. However, studies regarding the dermatome technique reported an overall higher rate of skin problems [35–37] compared to the linear incision technique [8, 19], although methodological variability could influence these outcomes and impair adequate comparison [16]. The linear incision technique is more and more used as the preferred technique in many clinics. Moreover, several promising modifications in this surgical approach are investigated in the current literature, for example the use of minimally invasive techniques without subcutaneous tissue thinning [20–23]. Unfortunately, some of these studies use the dermatome technique as control cohort [20, 21]. Nevertheless, to our knowledge, this is the first large-scale historical cohort study that actually directly compares patients operated with the dermatome and the linear incision technique with soft tissue reduction in the context of skin problems using the Holgers grading system consequently. It will contribute to more solid support of the linear incision technique as preferred surgical technique in the bone-anchored hearing implant surgery.

Furthermore, this study reveals a relatively long follow-up with a median of almost 4 years. The presence of skin problems is concentrated in the first years postoperatively, thus in most implants this period is covered. In addition, only four (2.9 %) of all identified implants in adults placed during the study period had to be excluded. The combination of this very low exclusion rate and a presence of (adverse) soft tissue reaction that is comparable with other studies, though for dermatome technique somewhat lower, [16] suggests a representative sample.

Moreover, both surgical techniques were performed by the same surgeon, so differences in other aspects of the surgical and perioperative approaches could be minimized to prevent possible confounding. In addition, this surgeon himself saw in general all patients during their complete follow-up. Regarding the subjective interpretation of most of the outcome measures, this small variability in observers is rather advantageous.

Nevertheless, the allocation of patients was not randomized. As stated, patients with one or more (suspected) risk factors for skin problems underwent implantation with the linear incision technique in most cases. Therefore, significantly more patients with risk factors (i.e. higher age, diabetes mellitus, cardiovascular disease and smoking) were seen in that cohort. This selection bias may have led to an underestimation of the skin problems in the dermatome cohort and overestimation in the linear incision group. Hence, there could have been a difference in (adverse) soft tissue reaction if there would have been a more equal distribution.

In addition, 91 of 132 included implants (68.9 %) were previous generation implants from Cochlear (“flange fixture”). This is a limitation of the study, because ongoing advances in implants and abutments have led to less skin reactions in the current types [38], with most recently, for example, the introduction and investigation of abutments with a hydroxyapatite coating [39, 40]. Our local Bone Implant database revealed that adverse soft tissue reactions Holgers ≥2 were significantly more encountered in the cohort with previous generation implants (“flange fixture”) compared to the newer implant abutments, i.e. Ponto Regular and BIA300. Although not significant, there was clearly a trend of less soft tissue reactions Holgers ≥1 and skin thickening in patients with these newer implants. The significantly higher rate of previous generation implants in the linear incision cohort contributes to the presumption that skin problems would have been noticed less frequently in this group, if the rate of current implant types was comparable with the dermatome group. Consequently, there may have been a difference in (adverse) soft tissue reaction and an even greater difference in presence of skin thickening.

An additional point of discussion is the missing of Holgers classification in, however, a substantial minority, of the follow-up contacts. In these cases, only a description of the skin surrounding the titanium skin-penetrating abutment was available and, as comprehensively described in Materials and Methods, assumptions were made about the presence or absence of a soft tissue reaction. Moreover, there does not exist a uniform grading system of skin thickening in the international literature yet. Nevertheless, as compared to other studies, the grade of skin thickening noticed was relatively mild. There was no overgrowth of skin reported and revision surgery was performed in only 2 patients (one from each group) [8, 11, 16, 35, 36].

As to speculate on possible causes for the higher rate of skin thickening following the dermatome technique, two factors might be of interest. First, the periost is preserved in the dermatome technique whereas removed in the linear incision approach, which might result in different mobility of the skin surrounding the abutment. Second, although both techniques make use of subcutaneous soft tissue reduction, the technical performance of this reduction (i.e. manually or mechanically) might be of influence in postoperative outcomes. In other words, skin reduction in the linear incision technique is less invasive and for that reason causes less traumatized skin, which would result in a lower percentage of patients with skin thickening.

In conclusion, no significant difference was found in the presence of soft tissue reactions and adverse soft tissue reactions (i.e. Holgers grade 2 or higher) between the dermatome and linear incision technique. However, the allocation of significantly more patients with risk factors and patients with previous generation implants to the linear incision cohort may have caused an underestimation of the difference between these two techniques. Skin thickening was significantly more seen in patients operated with the dermatome technique, which was treated successfully in all cases. Although items like aesthetic appearance, numbness, surgery time and healing time are not addressed in the current study, the linear incision technique should be preferred over the dermatome technique, based on the combination of no difference or possibly more (adverse) soft tissue reactions in the dermatome cohort and a significantly higher rate of skin thickening in this group.

As a matter of fact, this is the first historical cohort study directly comparing two widely used surgical techniques for BAHI implantation in such a large group of patients with a long-term follow-up. It adds knowledge for clinical practice and research and also contributes as a useful reference work. This study shows the strength of the linear incision in minimizing postoperative skin problems. Such well-founded evidence is of great importance, especially in the dynamic field of ongoing developments in bone-anchored hearing implants and surgical implantation techniques.
